# The Role of the Aging Bladder in Lower Urinary Tract Symptoms: Pathophysiology and Therapeutic Implications in Patients with Benign Prostatic Hyperplasia

**DOI:** 10.3390/medicina62040685

**Published:** 2026-04-03

**Authors:** Dimitrios Papanikolaou, Christos Diamantopoulos, Ioannis Sokolakis, Merkourios Kolvatzis, Georgios Antoniadis, Kyriakos Moysidis, Konstantinos Hatzimouratidis, Michael Samarinas

**Affiliations:** 1 General Hospital Papageorgiou, 2nd Urology Department, Aristotle University of Thessaloniki, 56429 Thessaloniki, Greecediamantopc@gmail.com (C.D.); sokolakisi@gmail.com (I.S.);; 2 Urology Department, General Hospital of Larissa, 41221 Larissa, Greece

**Keywords:** aging bladder, lower urinary tract symptoms (LUTS), benign prostatic hyperplasia (BPH), bladder ischemia, detrusor underactivity

## Abstract

*Background and Objectives*: Lower urinary tract symptoms (LUTS) are highly prevalent among aging men and have traditionally been attributed primarily to benign prostatic hyperplasia (BPH) and bladder outlet obstruction (BOO). However, growing evidence suggests that bladder-related mechanisms play a critical and often underrecognized role. This review aims to summarize current evidence on the contribution of the aging bladder to LUTS pathophysiology and to explore the therapeutic implications in men with BPH. *Materials and Methods*: A comprehensive literature search was conducted in PubMed/MEDLINE, Scopus, and Web of Science for studies published between January 2010 and April 2025. Search terms included combinations of “aging bladder”, “detrusor dysfunction”, “LUTS”, “BPH”, “bladder outlet obstruction”, “ischemia”, “overactive bladder”, and “detrusor underactivity”. Eligible studies included narrative reviews, systematic reviews, meta-analyses, clinical studies, and translational research addressing bladder aging and its clinical implications. A narrative synthesis approach was used due to heterogeneity in study design and outcomes. *Results*: A total of 43 studies were included in the qualitative synthesis. The evidence indicates that LUTS in older men result from multifactorial processes involving not only prostatic enlargement but also bladder dysfunction. Aging-associated changes include detrusor remodeling, impaired compliance, neural alterations, and vascular insufficiency, particularly chronic ischemia and oxidative stress. These mechanisms contribute to both detrusor overactivity and underactivity, providing a unifying framework for storage and voiding symptoms. Importantly, the severity of LUTS does not consistently correlate with prostate size or degree of obstruction. *Conclusions*: LUTS in aging men should be considered a complex condition involving both bladder and outlet factors. A bladder-centered perspective may improve patient stratification and therapeutic outcomes. Integrating bladder-targeted therapies with conventional BPH management supports a more personalized and effective approach to care.

## 1. Introduction

Lower urinary tract symptoms (LUTS) have a high prevalence in older men. 35% of men 60–69 years old and 90% of men 90 years old report LUTS [1]. Historically, benign prostatic hyperplasia (BPH) and its associated bladder outlet obstruction (BOO) have been considered the dominant causes of LUTS [1,2]. However, more recent data highlights a multifactorial pattern in aging-related changes in the bladder. Structural, neural, vascular, inflammatory, and microbiome factors play an important role in LUTS pathophysiology, alongside the prostate itself [3,4].

The relationship between prostate size and LUTS severity is inconsistent, and many patients exhibit persistent symptoms despite relief of obstruction, underscoring contributions beyond prostatic enlargement. Contemporary reviews and observational data show that LUTS have limited diagnostic specificity for BOO, and symptom burden does not reliably track with gland size [5]. Recent imaging-based and clinical studies likewise report discordance—some men have LUTS without significant enlargement, while others have enlargement without symptoms—supporting a broader, bladder-centered view of disease mechanisms [6].

Aging-associated bladder alterations encompass detrusor remodeling and compliance changes, efferent neural dysfunction, and particularly vascular mechanisms such as chronic pelvic/bladder ischemia and oxidative stress [7]. Evidence from translational and consensus reviews since 2010 links ischemia-induced oxidative injury to both detrusor overactive (DO) and detrusor underactive (DU) bladder phenotypes, providing a unifying framework for storage and voiding symptoms in the elderly [8]. These data suggest that progressive hypoxia, reactive oxygen species, and downstream inflammatory signaling contribute to fibrosis, denervation, and contractile impairment in the aging bladder [9,10].

These insights have therapeutic implications. While prostate-directed therapies remain essential for BOO, optimizing outcomes in older men with LUTS requires concurrent attention to bladder pathobiology. Growing clinical experience with bladder-targeted pharmacotherapies (e.g., β3-adrenoceptor agonists) and refined phenotyping of underactive and overactive bladder in older adults point toward a multimodal, personalized approach that addresses both outlet and detrusor factors [11].

## 2. Materials and Methods

**Figure 1:**
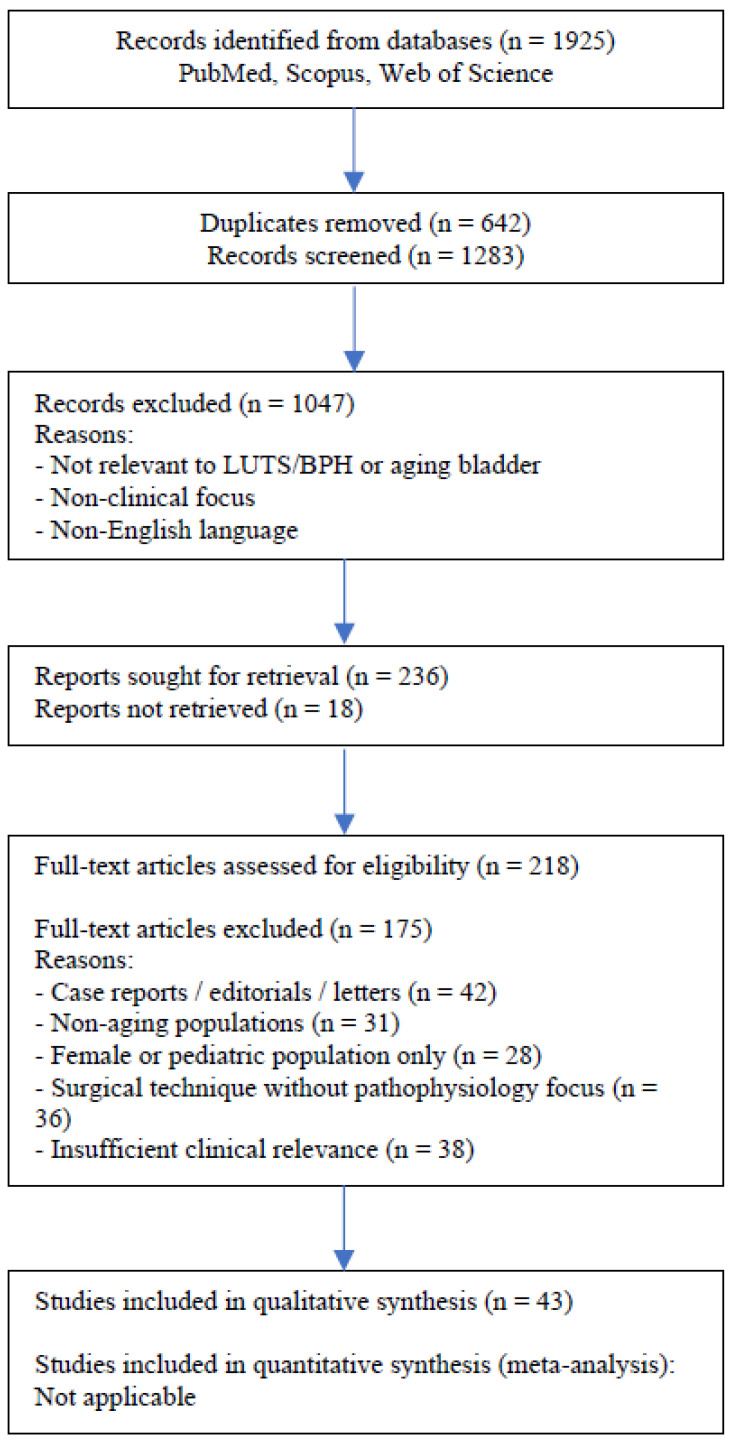
PRISMA 2020 diagram.

This review was conducted as a structured narrative review informed by systematic search principles and reported in accordance with the Preferred Reporting Items for Systematic Reviews and Meta-Analyses (PRISMA) framework, where applicable. Given the heterogeneity of study designs and outcomes, a formal systematic review with quantitative synthesis was not feasible. A total of 1925 records were identified through database searching. After removal of duplicates, 1283 records were screened based on title and abstract, of which 1047 were excluded. A total of 236 full-text articles were assessed for eligibility, and 175 were excluded based on predefined criteria. Ultimately, 43 studies were included in the qualitative synthesis. The detailed study selection process is presented in Figure 1.

A comprehensive literature search was performed in PubMed/MEDLINE, Scopus, and Web of Science for studies published between January 2010 and April 2025. The search strategy combined Medical Subject Headings (MeSH) and free-text terms, including but not limited to: “aging bladder,” “detrusor dysfunction,” “lower urinary tract symptoms,” “LUTS,” “benign prostatic hyperplasia,” “BPH,” “bladder outlet obstruction,” “ischemia,” “oxidative stress,” “overactive bladder,” and “detrusor underactivity.”

Study selection was performed in two stages. First, titles and abstracts were screened for relevance. Subsequently, full-text articles were assessed against predefined inclusion and exclusion criteria. Eligible studies included narrative and systematic reviews, meta-analyses, clinical trials, observational studies, and translational research with clear clinical implications. Studies focusing exclusively on non-aging populations, women, pediatric cohorts, or purely surgical techniques without pathophysiological relevance were excluded.

Due to the inclusion of heterogeneous study types, a standardized risk-of-bias assessment tool was not uniformly applicable. Instead, emphasis was placed on study design, methodological rigor, and consistency of findings across the literature. Evidence was synthesized narratively and organized into thematic domains focusing on pathophysiology and therapeutic implications.

## 3. Results

The literature search identified a heterogeneous body of evidence addressing the role of aging-related bladder dysfunction in the pathophysiology and management of lower urinary tract symptoms (LUTS) in men with benign prostatic hyperplasia (BPH). Due to variability in study design, populations, and outcomes, findings were synthesized narratively and are presented across two principal domains: (i) pathophysiological mechanisms and (ii) therapeutic implications.

A total of 43 studies met the inclusion criteria, including narrative and systematic reviews, clinical studies, and translational investigations. Overall, the evidence supports a multifactorial model of LUTS, in which bladder dysfunction contributes alongside bladder outlet obstruction (BOO). The main characteristics of the included studies are summarized in Table 1.

### 3.1. Study Characteristics

The included studies comprised a mix of clinical and experimental designs. Clinical studies primarily involved adult and elderly men with LUTS, often in the context of BPH, while translational studies provided mechanistic insights using animal models of bladder ischemia, obstruction, and aging.

Across study types, there was consistent agreement that LUTS severity does not correlate reliably with prostate size or degree of obstruction, supporting the contribution of non-prostatic factors, particularly bladder dysfunction.

### 3.2. Pathophysiological Findings

Across studies, aging-related bladder dysfunction emerged as a complex, progressive process involving structural, neural, vascular, and molecular alterations.

Structural remodeling of the detrusor muscle was a consistent finding, characterized by increased collagen deposition, reduced elasticity, and impaired compliance. These changes were associated with decreased contractile efficiency and contributed to both storage and voiding symptoms. Several studies highlighted that detrusor underactivity (DU) is highly prevalent in older men and often underrecognized, particularly in those with persistent symptoms following relief of obstruction [2,7,8,9,10].

Neurogenic alterations were also frequently reported. Aging was associated with degeneration of autonomic and sensory pathways, reduced neurotransmitter activity, and impaired urothelial signaling. These changes were linked to diminished bladder sensation and contractility in underactive bladder, as well as to increased afferent activity and urgency symptoms in earlier stages [9].

Vascular dysfunction and chronic bladder ischemia have emerged as important and biologically plausible contributors to aging-related bladder dysfunction. Experimental and translational studies suggest that reduced pelvic blood flow may lead to repeated cycles of hypoxia and reperfusion injury, promoting oxidative stress, mitochondrial dysfunction, and inflammatory activation [7,8].

However, it should be emphasized that much of the mechanistic evidence derives from animal models, translational studies, and consensus reports, and direct causal relationships in humans remain incompletely established. While clinical observations—such as the association between vascular disease and LUTS—support a potential role for ischemia, these findings should be interpreted with caution.

A commonly proposed model describes a biphasic response to ischemic injury, in which early stages are associated with detrusor overactivity, whereas prolonged or severe ischemia may lead to detrusor underactivity and fibrosis. Although this framework provides a useful conceptual model, it should be regarded as a hypothesis supported primarily by preclinical and indirect clinical evidence [7,8,9].

At the molecular level, oxidative stress and inflammation were central features. Increased production of reactive oxygen species, cytokine activation, and disruption of calcium signaling were shown to impair smooth muscle function and promote fibrosis. These processes provided a unifying mechanism linking aging, metabolic comorbidities, and bladder dysfunction [8].

The pathophysiology of the aging bladder is multifactorial and involves the interplay between vascular, molecular, neural, and structural mechanisms. Chronic ischemia appears to play a central role, linking systemic vascular disease to progressive bladder dysfunction through oxidative stress, inflammation, and tissue remodeling. These processes lead to a continuum of functional changes, ranging from detrusor overactivity in early stages to detrusor underactivity and fibrosis in advanced disease [7,8,9,10].

**Figure 2:**
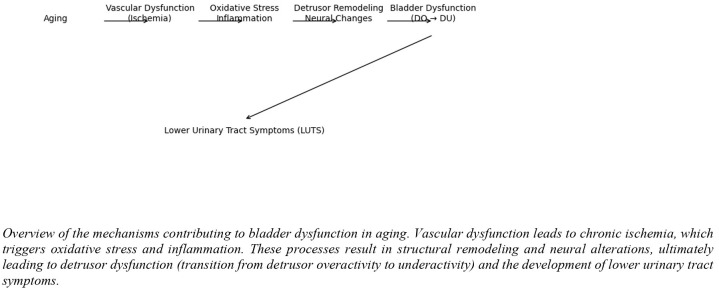
Pathophysiology of the aging bladder.

A schematic representation of these mechanisms is shown in Figure 2.

Collectively, these findings support a paradigm shift in which LUTS in aging men are not solely driven by prostate enlargement but frequently reflect primary bladder pathology.

The principal mechanisms of aging-related bladder dysfunction and their clinical implications are summarized in Table 2.

### 3.3. Therapeutic Findings

The reviewed literature highlights the importance of aligning treatment strategies with underlying pathophysiological mechanisms.

β3-adrenoceptor agonists were consistently shown to improve storage symptoms, with a favorable safety profile in elderly patients and minimal impact on detrusor contractility. These characteristics support their use in patients with predominant storage symptoms [11].

Antimuscarinic agents were also effective in reducing urgency and frequency; however, their use was associated with a higher incidence of adverse effects, particularly in older populations. Combination therapy with α-blockers may provide additional benefit in selected patients, although careful monitoring is required in those with elevated post-void residual (PVR) volumes or suspected detrusor underactivity [25,30].

Phosphodiesterase type 5 (PDE5) inhibitors demonstrated modest improvements in LUTS in randomized trials and meta-analyses. Their proposed mechanisms include enhancement of nitric oxide signaling and improvement of pelvic perfusion. However, the translation of these mechanisms into clearly defined clinical phenotypes remains limited, and their role should be considered adjunctive rather than primary [7,8,25].

Overall, the evidence supports a multimodal, individualized approach, integrating both bladder-directed and outlet-directed therapies based on symptom profile and underlying dysfunction [25].

A summary of available treatment options, their mechanisms of action, and clinical considerations is provided in Table 3.

### 3.4. Surgical Outcomes

Surgical treatment effectively reduces bladder outlet resistance and improves urinary flow in appropriately selected patients. However, outcomes are variable, particularly in older individuals.

A consistent finding across studies is that surgical intervention does not reverse established bladder dysfunction. Patients with preserved detrusor contractility derive the greatest benefit, whereas those with detrusor underactivity are more likely to experience persistent symptoms or incomplete bladder emptying [25,30].

Several urodynamic parameters, including bladder contractility index and bladder compliance, were identified as predictors of surgical success. In contrast, elevated PVR and impaired contractility were associated with poorer outcomes [2,3].

These findings highlight the importance of appropriate patient selection and suggest a role for urodynamic assessment in selected cases, particularly when the diagnosis is uncertain or when surgical outcomes are difficult to predict [25].

### 3.5. Summary of Evidence

Collectively, the evidence indicates that LUTS in aging men arise from the interplay between bladder dysfunction and bladder outlet obstruction. Bladder-related mechanisms—including structural remodeling, neural alterations, and vascular changes—contribute significantly to symptom development and treatment response.

These findings support a shift toward a mechanism-based and phenotype-oriented approach, in which evaluation and management are tailored to the relative contribution of bladder and outlet factors.

**Figure 3:**
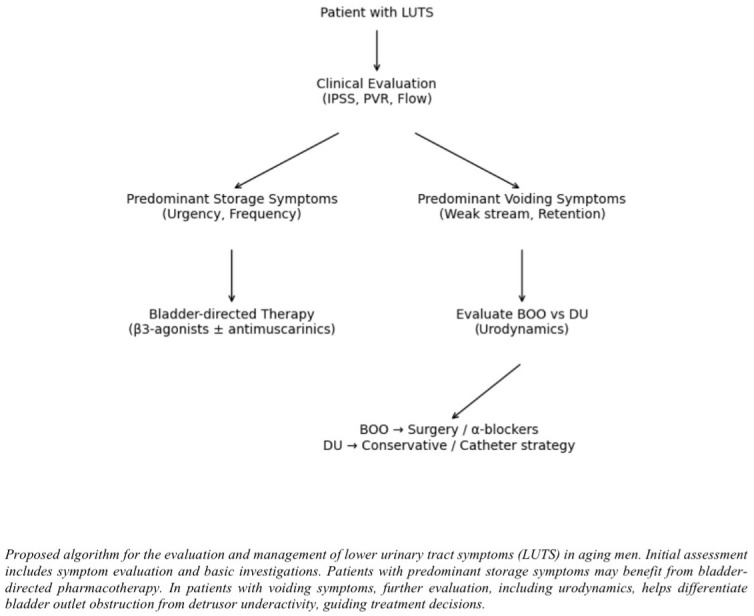
Clinical approach to LUTS in the aging bladder.

Based on current evidence, a practical approach to the evaluation and management of LUTS in aging men is proposed in Figure 3.

## 4. Discussion

The present review highlights the evolving understanding of lower urinary tract symptoms (LUTS) in aging men, emphasizing that these symptoms cannot be adequately explained by bladder outlet obstruction (BOO) alone. Instead, accumulating evidence supports a multifactorial model, in which intrinsic bladder dysfunction driven by aging-related structural, neural, vascular, and molecular alterations plays a central role. This paradigm shift has important implications for both diagnosis and management.

### 4.1. From a Prostate-Centric to a Bladder-Centric Model

Historically, LUTS in men have been attributed primarily to benign prostatic hyperplasia (BPH) and its associated obstruction. However, several clinical and epidemiological observations challenge this paradigm. Symptom severity correlates poorly with prostate size, and a substantial proportion of patients continue to experience LUTS despite adequate relief of obstruction [25,30]. Furthermore, imaging and urodynamic studies have demonstrated that LUTS frequently occur in the absence of significant enlargement, while conversely, some men with marked enlargement remain asymptomatic [6].

These discrepancies underscore the limited specificity of LUTS for diagnosing BOO and point toward a broader pathophysiological framework. Contemporary consensus statements emphasize that LUTS represent a syndrome rather than a disease entity, encompassing storage, voiding, and post-micturition symptoms arising from both bladder and outlet dysfunction [30,31]. In this context, the aging bladder emerges as a key determinant of symptom generation and treatment response.

### 4.2. The Aging Bladder as a Unifying Pathophysiological Concept

Aging-related bladder dysfunction is the result of cumulative insults affecting the detrusor muscle, its innervation, and its vascular supply. These processes interact dynamically and evolve over time, producing a spectrum of clinical phenotypes.

Structural remodeling of the detrusor, characterized by smooth muscle loss, collagen deposition, and reduced elasticity, leads to impaired compliance and contractility [3,16]. These changes contribute to both storage symptoms, through decreased accommodation, and voiding symptoms, through impaired emptying. Importantly, once fibrosis is established, these changes may be irreversible, explaining the persistence of symptoms after surgical relief of obstruction.

Neurogenic alterations further compound these effects. Aging is associated with degeneration of both afferent and efferent pathways, altered neurotransmitter release, and impaired receptor sensitivity [32]. Reduced cholinergic signaling contributes to detrusor underactivity (DU), while afferent sensitization may lead to urgency and detrusor overactivity (DO) in earlier stages. The urothelium, now recognized as an active sensory organ, also exhibits age-related dysfunction, further disrupting bladder reflexes [7,9,13].

Among the various mechanisms, chronic bladder ischemia has emerged as a central and unifying factor. Age-related vascular disease, endothelial dysfunction, and atherosclerosis reduce pelvic perfusion, exposing the bladder to repeated hypoxia–reperfusion injury [9,33]. Experimental models consistently demonstrate that ischemia induces oxidative stress, mitochondrial dysfunction, and inflammatory activation, leading to progressive structural and functional impairment [7,8].

A particularly important concept is the biphasic response to ischemia. Early ischemic injury is associated with increased afferent activity and detrusor overactivity, whereas chronic or severe ischemia leads to smooth muscle degeneration, fibrosis, and detrusor underactivity [10,12]. This temporal progression provides a mechanistic explanation for the clinical observation that patients may transition from storage-predominant symptoms to voiding dysfunction over time.

At the molecular level, oxidative stress and chronic inflammation represent key mediators of bladder aging. Increased production of reactive oxygen species, activation of pro-inflammatory cytokines, and dysregulation of calcium signaling impair excitation–contraction coupling and promote fibrosis [8]. These processes link bladder dysfunction to systemic conditions such as metabolic syndrome and cardiovascular disease, which are highly prevalent in aging populations [9,14,34].

### 4.3. Clinical Implications: Toward Phenotype-Driven Management

The recognition of bladder dysfunction as a key contributor to LUTS supports a phenotype-driven and bladder-centered approach to evaluation and management. This approach is particularly relevant in specific clinical scenarios where standard prostate-centered strategies may be insufficient.

Bladder-focused assessment should be considered in patients with persistent LUTS despite adequate relief of bladder outlet obstruction, individuals with low maximum flow rate and elevated post-void residual, cases with suspected detrusor underactivity and elderly or frail patients, in whom treatment goals prioritize quality of life and functional outcomes.

In routine practice, uroflowmetry and PVR measurement represent useful initial tools to identify patients with impaired voiding efficiency. In selected cases, particularly when the diagnosis is uncertain or before surgical intervention, urodynamic studies can help distinguish between bladder outlet obstruction and detrusor dysfunction.

These distinctions have direct therapeutic implications. Patients with preserved detrusor contractility and clear evidence of obstruction are more likely to benefit from surgical intervention. In contrast, those with significant DU may experience limited improvement after de-obstruction and may be better managed with conservative or bladder-directed strategies.

In patients with predominant storage symptoms, particularly in the absence of significant obstruction, bladder-targeted pharmacotherapy (e.g., β3-adrenoceptor agonists) may be preferred. Conversely, patients with mixed symptom profiles may benefit from combination approaches.

In frail elderly patients, treatment goals should be individualized, with emphasis on symptom relief, avoidance of adverse effects, and maintenance of independence, rather than normalization of urodynamic parameters.

For improved clinical applicability, commonly used thresholds are provided as guiding values: prostate volume enlargement is typically considered at >30–40 mL (imaging-dependent); post-void residual at 100 mL suggestive of impaired bladder emptying, while at 300 mL associated with increased risk of urinary retention and poorer surgical outcomes; maximum flow rate < 15 mL/sec is considered abnormal, while <10 mL/s is strongly suggestive of bladder outlet obstruction or detrusor underactivity. These thresholds are not absolute and should be integrated with symptom profile and additional testing [2,31].

The International Prostate Symptom Score (IPSS) provides a practical framework for symptom characterization, separating storage symptoms (frequency, urgency, nocturia) from voiding symptoms (weak stream, intermittency, hesitancy, incomplete emptying). However, the correlation between IPSS subdomains and urodynamic findings is imperfect. Several studies demonstrate that symptom profiles alone cannot reliably distinguish between obstruction and impaired contractility, reinforcing the role of urodynamics in selected patients [1,2,25,30,31].

### 4.4. Epidemiological and Outcomes Data—Preoperative Work-Up

Clinical and observational data indicate that a proportion of patients experience persistent or recurrent LUTS after surgical de-obstruction, particularly in the presence of underlying bladder dysfunction. Reported rates of persistent symptoms vary across studies, reflecting differences in patient selection and baseline characteristics [2,7].

Patients with detrusor underactivity, elevated PVR, or poor contractility indices are consistently shown to have less favorable outcomes following procedures such as TURP. These findings support the importance of appropriate preoperative evaluation, particularly in patients with atypical symptoms or suspected bladder dysfunction [7,16,25,30,31].

### 4.5. Medical Therapy: Aligning Treatment with Pathophysiology

The evolving understanding of bladder aging has important implications for pharmacological management. Traditional therapies targeting the prostate may be insufficient in patients with significant bladder dysfunction, highlighting the need for bladder-directed treatments.

β3-adrenoceptor agonists have emerged as a particularly suitable option for the aging bladder. By promoting detrusor relaxation during the storage phase without impairing contractility, they effectively address urgency and frequency while preserving voiding function. Their favorable safety profile, particularly with respect to cognitive adverse effects, makes them especially attractive in older populations [11,17,35].

In contrast, antimuscarinic agents, while effective for storage symptoms, are limited by systemic adverse effects, including dry mouth, constipation, and cognitive impairment [36]. These risks are particularly relevant in elderly patients with polypharmacy and frailty [18,19,20]. Consequently, their use should be individualized and carefully monitored.

Phosphodiesterase type 5 (PDE5) inhibitors have demonstrated modest improvements in LUTS in randomized trials and meta-analyses. Their proposed mechanisms include enhancement of nitric oxide signaling, improvement of pelvic blood flow, and modulation of afferent nerve activity [21,22,37].

However, the translation of these mechanistic effects into a clearly defined clinical phenotype remains limited. The relationship between vascular mechanisms and treatment response is not yet sufficiently characterized to allow targeted patient selection.

Therefore, PDE5 inhibitors should be considered as a potential adjunctive option, particularly in patients with concomitant erectile dysfunction, rather than as a primary mechanism-driven therapy [23,38,39,40].

### 4.6. Surgical Outcomes and the Limits of De-Obstruction

Surgical management remains the cornerstone of treatment for patients with significant BOO. Procedures such as transurethral resection of the prostate (TURP) and laser enucleation effectively reduce outlet resistance and improve urinary flow [24,41]. However, their impact on symptoms is highly variable, particularly in older patients.

A key limitation of surgical intervention is that it does not reverse established bladder dysfunction. Patients with preserved detrusor contractility derive the greatest benefit, whereas those with DU often have suboptimal outcomes, including persistent symptoms and continued need for catheterization [15]. These findings underscore the importance of preoperative assessment of bladder function [42].

Urodynamic evaluation can improve patient selection and help predict outcomes. Patients with demonstrable obstruction and adequate contractility are more likely to benefit from surgery, whereas those with severe DU may require alternative or adjunctive management strategies [16,26,27,28]. In this context, surgery should be viewed as one component of a broader, multimodal approach rather than a definitive solution [29].

### 4.7. Bladder Health Preservation and the “Window of Curability”

Recent conceptual frameworks have emphasized a shift from a prostate-centered to a bladder health–preserving approach in men with LUTS attributed to BPH/BPO. The bladder, as an end organ with limited regenerative capacity, may undergo progressive and potentially irreversible dysfunction if obstruction is not addressed in a timely manner. In this context, the concept of a “window of curability” has been proposed, suggesting that early identification and intervention may prevent or delay the transition to advanced stages of bladder dysfunction [43].

A staging model describing the evolution of bladder health in BPH/BPO has been introduced, encompassing a continuum from bladder outlet obstruction to detrusor overactivity, urgency incontinence, acute urinary retention, and ultimately detrusor underactivity. While transitions between stages are not necessarily linear and may overlap, this framework highlights the potential consequences of delayed intervention and underscores the importance of timely, individualized management [43,44,45].

Although this model remains to be validated in prospective clinical studies, it provides a useful conceptual tool for risk stratification, patient counseling, and shared decision-making, aligning with a more proactive and mechanism-based approach to LUTS management.

### 4.8. Limitations of Current Evidence

Despite growing interest in the aging bladder, several limitations remain. First, there is significant heterogeneity in study design, patient populations, and outcome measures, which complicates the interpretation and comparison of results. Second, the definition and diagnosis of detrusor underactivity remain inconsistent, limiting the ability to standardize research and clinical practice [29,46].

Most available data are derived from observational studies and animal models, with relatively few prospective clinical trials specifically addressing bladder dysfunction in aging populations. Furthermore, the complex interplay between bladder, prostate, and systemic factors is not fully understood, and causal relationships remain difficult to establish.

### 4.9. Future Directions

Future research should focus on improving the characterization of bladder dysfunction in aging populations. The development of non-invasive biomarkers, advanced imaging techniques, and refined urodynamic parameters may facilitate earlier diagnosis and more precise phenotyping.

There is also increasing interest in therapies targeting the underlying mechanisms of bladder aging, including agents that reduce oxidative stress, improve vascular function, or promote tissue regeneration. Experimental studies suggest that antioxidant and anti-inflammatory strategies may have therapeutic potential, although clinical data are currently limited [10].

Ultimately, the integration of clinical, urodynamic, and molecular data may enable a precision medicine approach to LUTS, in which treatment is tailored to the individual patient’s pathophysiology.

## 5. Conclusions

The aging bladder plays a central role in the pathogenesis of LUTS in men and represents a key determinant of treatment outcomes. While bladder outlet obstruction remains an important contributor, it is increasingly clear that LUTS reflect a complex interplay between outlet and detrusor factors.

A shift toward a bladder-centered, mechanism-based approach is essential for optimizing management, particularly in older patients. This includes careful patient phenotyping, judicious use of urodynamics, and individualized treatment strategies that address both obstruction and bladder dysfunction.

Recognizing the aging bladder as a primary driver of LUTS provides a more comprehensive framework for understanding disease progression and offers new opportunities for therapeutic innovation.

## Figures and Tables

**Table 1:** Key studies on aging bladder and LUTS in men with BPH.

Study (First Author, Year)	Study Type	Population/Model	Key Focus	Main Findings	Clinical Relevance
Parsons, 2010 [1]	Narrative review	Epidemiological data	LUTS prevalence	High prevalence of LUTS increases with age	Supports burden of disease in elderly men
Roehrborn, 2011 [2]	Review	Clinical data	BPH and LUTS	BOO is important but not sole cause of LUTS	Introduces limitations of prostate-centric model
Jung, 2015 [3]	Review	Elderly patients	Geriatric LUT dysfunction	Multifactorial causes including bladder dysfunction	Emphasizes aging-related changes
Lee, 2017 [4,5]	Review	Clinical studies	Diagnosis of LUTS/BPH	Poor correlation between prostate size and symptoms	Supports need for broader evaluation
Moryousef, 2023 [6]	Observational imaging study	Men with LUTS	MRI prostate parameters	Discordance between prostate size and symptoms	Confirms weak size–symptom relationship
Speich, 2020 [7]	Consensus report (ICI-RS)	Translational + clinical	Ischemia & oxidative stress	Ischemia contributes to LUT dysfunction	Establishes vascular hypothesis
Yang, 2021 [8]	Translational study	Animal model	Bladder ischemia	Oxidative stress damages bladder tissue	Mechanistic insight
Andersson, 2022 [9]	Review	Experimental & clinical	Oxidative stress	ROS linked to LUTS	Unifying molecular pathway
Wu, 2021 [10]	Review	Animal + clinical	Ischemia-induced OAB	Ischemia causes DO and DU	Explains symptom progression
Zhao, 2016 [12]	Experimental study	Animal model	Chronic ischemia	Biphasic response: DO → DU	Key concept in aging bladder
Andersson, 2017 [13]	Review	Experimental & clinical	Vascular aging	Reduced perfusion leads to dysfunction	Links LUTS to systemic vascular disease
Papaefstathiou, 2023 [14]	Clinical study	Patients with vascular disease	Pelvic ischemia	Revascularization improves LUTS	Human evidence for ischemia role
Osman, 2018 [15]	Systematic review	Clinical + preclinical	Detrusor underactivity	DU is common and underdiagnosed	Important for treatment selection
van Koeveringe, 2011 [16]	Review	Clinical data	DU definition	Need for better characterization	Highlights diagnostic challenges
Makhani, 2020 [11]	Clinical study	Elderly OAB patients	β3-agonists	Effective and safe in elderly	First-line option for storage symptoms
O’Kane, 2022 [17]	Review	Clinical trials	Mirabegron	Good efficacy, low cognitive risk	Preferred in elderly
Usmani, 2019 [18]	Meta-analysis	Older vs. younger patients	Antimuscarinics	Higher adverse events in elderly	Safety concerns
Kaplan, 2011 [19]	Systematic review	Men with LUTS	Antimuscarinics	Effective but risk of retention	Use with caution
Lenfant, 2023 [20]	Meta-analysis	LUTS/BPH patients	Combination therapy	α-blocker + antimuscarinic improves symptoms	Combination approach
Gacci, 2016 [21]	Systematic review	LUTS/BPH patients	PDE5 inhibitors	Improve LUTS independent of prostate size	Mechanism-based therapy
Giuliano, 2013 [22]	Review	Experimental	PDE5 mechanism	Improves perfusion & signaling	Supports vascular hypothesis
Cui, 2021 [23]	Meta-analysis	Clinical trials	Tadalafil	Improves LUTS and QoL	Evidence-based therapy
Oelke, 2013 [24]	Guidelines (EAU)	Clinical data	LUTS management	Multimodal treatment approach	Clinical standard
Abrams, 2013 [25]	Guideline/consensus	Older men	LUTS evaluation	Importance of symptom assessment	Guides clinical practice
Antoniou, 2023 [26]	Observational study	Elderly TURP patients	Surgical outcomes	Variable outcomes in elderly	Importance of selection
Zou, 2024 [27]	Systematic review	DU patients	TURP outcomes	Limited benefit in DU	Highlights role of bladder function
Yang, 2023 [28]	Clinical study	TURP patients	Urodynamics	Contractility predicts outcomes	Supports preoperative testing
Wei, 2025 [29]	Review	General population	LUTS overview	Multifactorial nature of LUTS	Modern conceptual framework

**Table 2:** Pathophysiological mechanisms of aging bladder and their clinical implications.

Mechanism	Pathophysiology	Clinical Impact
**Structural remodeling**	Fibrosis, reduced compliance, impaired contractility	DU, incomplete emptying
**Neurogenic alterations**	Impaired signaling, reduced sensation, altered neurotransmission	DO or DU, urgency or weak stream
**Vascular dysfunction**	Reduced blood flow, chronic ischemia	Progressive bladder dysfunction
**Oxidative stress**	ROS production, mitochondrial dysfunction	Worsening contractility
**Inflammation**	Cytokine activation, tissue damage	Fibrosis and symptom progression

DO: detrusor overactivity, DU: detrusor underactivity, ROS: reactive oxygen species.

**Table 3:** Mechanism-based treatment strategies for LUTS in the aging bladder.

Therapy	Target Mechanism	Clinical Effect	Advantages	Limitations
**β3-adrenoceptor agonists**	Enhance detrusor relaxation during storage	Reduce urgency and frequency without impairing voiding	Low risk of cognitive effects; safe in elderly	Cost; slower onset
**Antimuscarinics**	Inhibit muscarinic receptor-mediated contractions	Reduce urgency and frequency	Effective for storage symptoms	Cognitive and systemic side effects in elderly
**PDE5 inhibitors**	Improve NO-cGMP signaling and pelvic perfusion	Improve both storage and voiding symptoms	Addresses vascular and sensory pathways	Cardiovascular considerations
**α-blockers**	Reduce bladder outlet resistance	Improve flow and voiding symptoms	Rapid symptom relief	Limited effect on storage symptoms
**Surgical treatment (TURP, laser enucleation)**	Relieve bladder outlet obstruction	Improve flow; variable symptom relief	Definitive treatment for BOO	Does not treat bladder dysfunction; variable outcomes

LUTS: lower urinary tract symptoms, PDE5: phosphodiesterase type 5, TURP: transurethral resection of prostate, BOO: bladder outlet obstraction.

## Data Availability

No new data were created or analyzed in this study.
